# New endemic familial parkinsonism in south Moravia, Czech Republic and its genetical background

**DOI:** 10.1097/MD.0000000000012313

**Published:** 2018-09-21

**Authors:** Tereza Bartoníková, Kateřina Menšíková, Kristýna Kolaříková, Radek Vodička, Radek Vrtěl, Pavel Otruba, Michaela Kaiserová, Miroslav Vaštík, Lenka Mikulicová, Josef Ovečka, Ludmila Šáchová, František Dvorský, Jiří Krša, Petr Jugas, Marek Godava, Martin Bareš, Vladimír Janout, Petr Hluštík, Martin Procházka, Petr Kaňovský

**Affiliations:** a Department of Neurology; b Department of Medical Genetics, Faculty of Medicine and Dentistry, Palacký University, University Hospital, Olomouc; c General Practitioner, Lipov; d General Practitioner, Velká nad Veličkou; e General Practitioner, Blatnice pod Svatým Antonínkem; f Neurology Outpatient Clinic, Veselí nad Moravou; g First Department of Neurology, Masaryk University Medical School, St. Anne University Hospital, Brno; h Department of Epidemiology and Public Health, Faculty of Medicine and Dentistry, Palacky University, University Hospital, Olomouc, Czech Republic.

**Keywords:** familial neurodegenerative parkinsonism, molecular-genetic background, population with long-lasting inbreeding behavior

## Abstract

An increased prevalence of familial neurodegenerative parkinsonism or cognitive deterioration was recently found in a small region of southeastern Moravia.

The aim of the study was to assess the genetic background of this familial disease.

Variants in the *ADH1C*, *EIF4G1*, *FBXO7*, *GBA + GBAP1*, *GIGYF2*, *HTRA2*, *LRRK2*, *MAPT*, *PRKN, DJ-1, PINK1*, *PLA2G6*, *SNCA*, *UCHL1*, *VPS35* genes were examined in 12 clinically positive probands of the pedigree in which familial atypical neurodegenerative parkinsonism was identified in previous epidemiological studies. Libraries were sequenced by massive parallel sequencing (MPS) on the Personal Genome Machine (PGM; Ion Torrent). Data were analyzed using Torrent Suite and IonReporter software. All variants were then verified and confirmed by Sanger sequencing.

We identified 31 rare heterozygous variants: 11 missense variants, 3 synonymous variants, 8 variants in the UTR region, and 9 intronic variants. Six variants (rs1801334, rs33995883, rs35507033, rs781737269, rs779760087, and rs63750072) were evaluated as pathogenic by at least one in-silico predictor.

No single “founder” pathogenic variant associated with parkinsonism has been found in any of the probands from researched pedigree. It may rather be assumed that the familial occurrence of this disease is caused by the combined influence of several “small-effect” genetic variants that accumulate in the population with long-lasting inbreeding behavior.

## 1 Introduction

The results of epidemiological studies indicate that the prevalence of Parkinson disease (PD) and neurodegenerative parkinsonism in the general population is around 1.6% in Europe and approximately 1.5% in Asia and the Americas.^[1–8]^ In the population over 65 years of age, the prevalence of these diseases ranges from 1.1% to 2.2%^[1–11]^ and even higher prevalences have been repeatedly found in small, isolated populations.^[3,4,6,9]^

**Figure 1:**
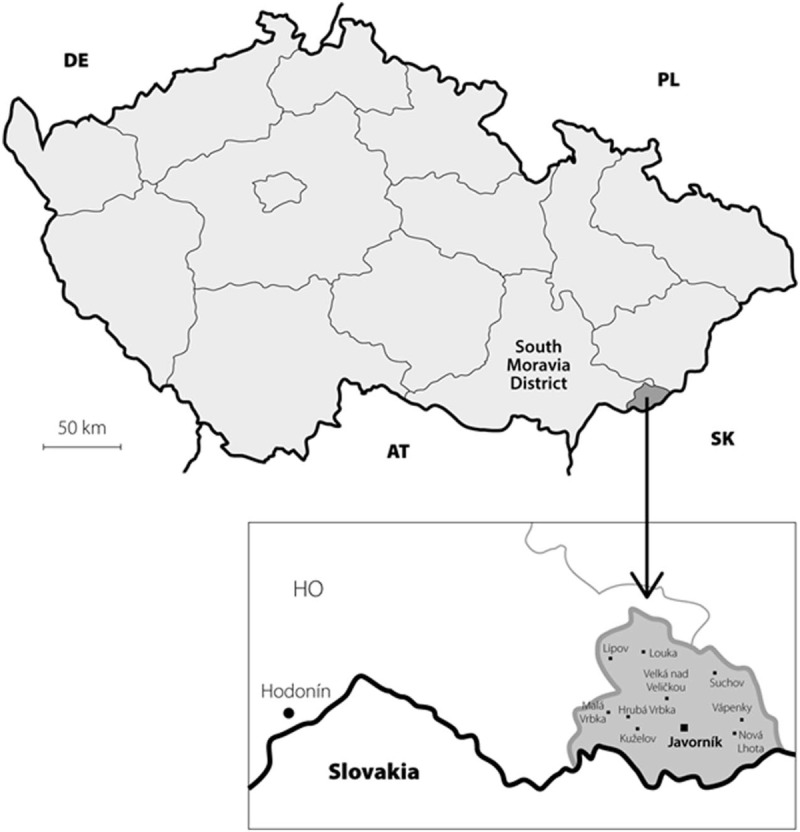
Map of Czech Republic with its district borders. The dark gray area in the lower right is the researched region with its 10 villages. The village where the researched pedigree originated (Javorník) is highlighted. DE: Germany, PL: Poland, AT: Austria, SK: Slovakia, HO: Hodonin county.

**Figure 2:**
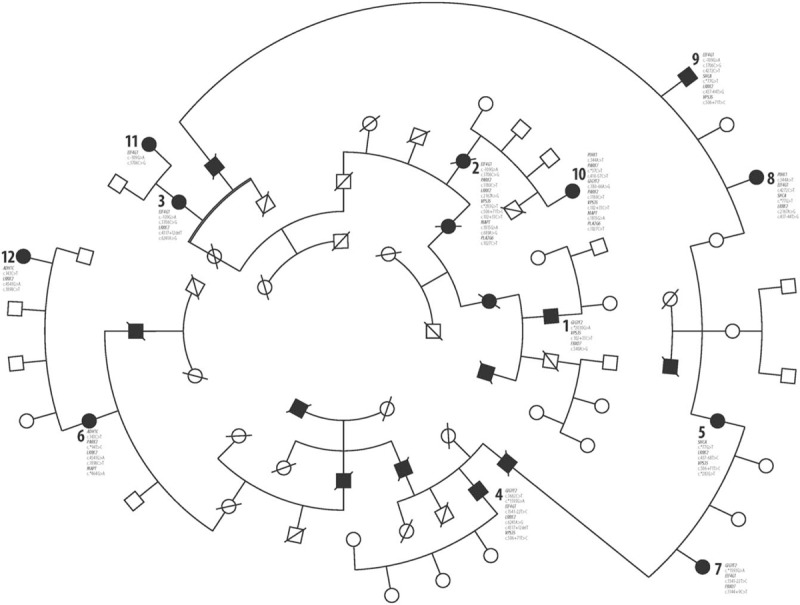
Large pedigree with familial parkinsonism and variants that have been identified in 12 symptomatic probands. The numbers of probands (1; 2; 3; etc.) in pedigree correspond with the numbers of probands in Table 1. The names of genes and their mutations which have been identified in individual probands are in the figure assigned to their numbers.

We recently described such a phenomenon in 10 villages of a remote, small, rural, and isolated Moravian region of Czech Republic called “Hornacko” [Upper Lands]. The last census, conducted in 2011, determined the presence of 8664 inhabitants, 2927 of which were over 50 years of age. “Hornacko” people have their own specific customs and traditions, which are reflected in the folk art, crafts, architecture, costumes, songs, and dances; also, the local dialect is very different from that of the surrounding areas (Fig. 1). This area is traditionally isolated from the neighboring Moravian regions and the people only rarely migrate permanently out of here.^[12,13]^ In 2011 to 2015, we conducted an epidemiological study in this region which has found a significantly increased prevalence of neurodegenerative parkinsonism. The overall prevalence in the population over 50 years of age was 2.8% (95% confidence interval (CI), 2.2–3.4); the prevalence in the population between 50 and 64 years of age was 1.9% (95% CI, 1.2–2.5) and it was 4.06% (95% CI, 2.9–5.1) in the population over 65 years of age.^[14,15]^ Three large pedigrees with a familial occurrence of parkinsonism were found in which clinical geneticists identified an autosomal-dominant inheritance pattern.^[16]^ Two of these pedigrees eventually compiled one large family tree spanning several generations from 1840 to the present.^[17]^ In this large pedigree, 12 probands with apparent clinical signs of parkinsonism were identified (Fig. 2). These probands were referred to a tertiary movement disorders center for detailed clinical and laboratory examinations including DNA sampling in order to obtain information about the clinical, biological, and genetic characteristics of this endemic and familial disorder.

The village from which these families originate (Javorník nad Veličkou, PCN CZ-69674, elevation 412 m, population 720) is very specific even in this region: the population belongs almost entirely to the Lutheran denomination, unlike all neighboring villages, which are entirely Roman-Catholic. Marriages between the inhabitants of Javorník and residents of other villages were and still are quite exceptional, so the local population is practically isolated from a genetic point of view.^[18]^

## 2 Methods

The study was approved by the ethics committee of Palacký University Medical School, and all patients and probands gave their informed consent.

All 12 probands who were evaluated as positive for parkinsonian symptoms were examined by a movement disorders specialist. The United Kingdom Parkinson's Disease society Brain Bank clinical diagnostic criteria clinical diagnostic criteria were used for the diagnosis of parkinsonism; this was diagnosed when at least 2 of 4 Class I symptoms (resting tremor, rigidity, bradykinesia, and impaired postural reflexes) were present in a subject not receiving antiparkinsonian medication. Cognitive functions were assessed using the Mini Mental State Examination (MMSE) tool; cognitive deterioration was diagnosed when the MMSE score was ≤24. All positive probands were then examined in detail to exclude secondary forms of parkinsonism. The examination protocol included detailed neurological, psychological, and psychiatric examinations, examination of cerebrospinal fluid, magnetic resonance imaging of the brain; neurophysiological examinations were also performed (electroencephalographic recordings, electromyography, and assessments of visual, auditory, somatosensory, and motor evoked potentials).^[16,17]^ The clinical details of all probands are presented in Table 1.

**Table 1:**
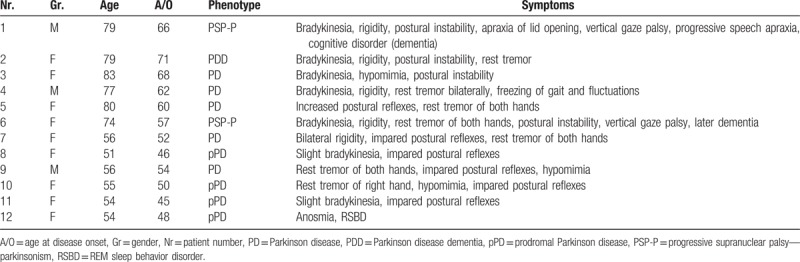
Clinical details of probands with clinical signs of parkinsonism.

DNA was isolated from peripheral blood by a DNA silica-gel membrane based isolation kit (QIAamp DNA Mini Kit - Qiagene; Strasse 1, 40724 Hilden, Germany). Each DNA sample was diluted to a final concentration of 10 ng/μL. The amplicon library was in silico designed by Ion AmpliSeq Designer (Thermo Fisher Scientific) for *ADH1C*, *EIF4G1*, *FBXO7*, *GBA + GBAP1*, *GIGYF2*, *HTRA2*, *LRRK2*, *MAPT*, *PRKN, DJ-1, PINK1*, *PLA2G6*, *SNCA*, *UCHL1*, and *VPS35* genes. In total, 617 amplicons covered 92.59% of Coding Sequence (Coding Region of a Gene) including 100 bp in the introns and Untranslated Regions of mRNA of the regions. Libraries were prepared using Ion AmpliSeq Library Kit 2.0 (Ion Torrent; Termo Fisher Scientific, Waltham, Massachusetts, USA), emulsion PCR was done on the Ion OneTouch 2 Instrument (Ion Torrent) with Ion PGM Template OT2 200 Kit. Samples were barcoded to enable loading 8 samples on one Ion 316 Chip. The amplicons were sequenced by massive parallel sequencing (MPS) on the Personal Genome Machine (PGM; Ion Torrent) using Ion PGM Sequencing Kit (Ion Torrent) and Ion 316 Chip Kit v2. For the coverage control were the amplicons checked in Torrent Suite (Termo Fisher Scientific, Waltham, Massachusetts, USA) using plugin Coverage Analysis. Data analysis and variant searching (Mapping, Variant calling, Annotation) was done by Torrent Suite and IonReporter software (Termo Fisher Scientific, Waltham, Massachusetts, USA). Data from PGM were collected and converted to Fastq and BAM formats using Ion Torrent Suite software. The Bam/Bai files were reloaded to IonReporter and variant calling workflow was in the first level relaxed to minimal amplicon coverage of 4 and a minimal ratio of alternative/common variant of 0.1. Variants were then filtered using the parameter of minor allele frequency (MAF) <0.01. All variants were then verified and confirmed by Sanger sequencing. SIFT and PolyPhen-2 prediction software were used for missense variant evaluation. The PhyloP algorithm was used to assess the phylogenetical conservation.

## 3 Results

The variants identified in our 12 probands are shown in Table 2; 31 rare heterozygous variants were identified.

**Table 2:**
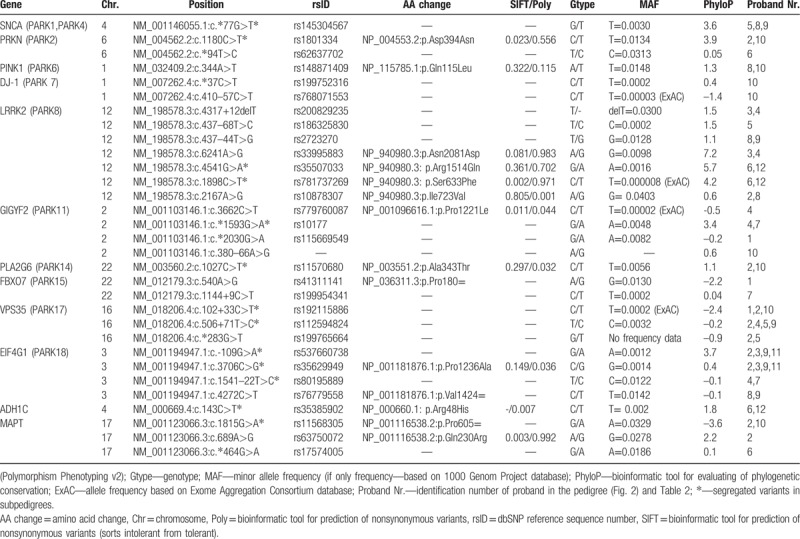
Genetic variants identified in probands with clinical signs of parkinsonism.

One variant was detected in the UTR-3 region in the *SNCA* (*PARK1*, *PARK4*) gene—c.∗77G>T (5,8,9). In the *PRKN (PARK2)* gene, we found 2 variants, 1 in the UTR-3 region—c.∗94T>C (6)—and 1 in exon—c.1180C>T (2,10). In the *PINK1* (*PARK6*) gene was found 1 variant in the coding sequence—c.344A>T (8,10). In *DJ-1* (*PARK7*) were found 2 variants in the noncoding region—c.∗37C>T (10) and c.410–57C>T (10). Three intronic—c.4317+12delT (3,4), c.437–68T>C (5), and c.437–44T>G (8,9)—and 4 exonic variants—c.2167A>G (2,8), c.6241A>G (3,4), c.4541G>A (6,12), and c.1898C>T (6,12) in the *LRRK2* (*PARK8*) gene were detected. In the *GIGYF2* (*PARK11*) gene were found 3 variants in the noncoding sequence—c.∗2030G>A (1), c.∗1593G>A (4,7), and c.380–66A>G (10) (this one has not been described before) and one in the coding sequence—c.3662C>T (4). In the *PLA2G6* (*PARK14*) gene, one exonic variant—c.1027C>T (2,10) was found. In *FBXO7*(*PARK15*), 2 variants—c.540A>G (1) and c.1144+9C>T (7) were detected. In *VPS35*(*PARK17*), 3 variants in the noncoding sequence—c.∗283G>T (2,5), c.102+33C>T (1,2,10), and c.506+71T>C (2,4,5,9) were detected. Among 4 variants, 2 in the noncoding sequence—c.109G>A (2,3,9,11), c.1541–22T>C (4,7)—and 2 in the coding sequence—c.3706C>G (2,3,9,11), c.4272C>T (8,9)—were detected in the *EIF4G1* (*PARK18*) gene. In the *ADH1C* gene was identified one variant located in the coding sequence—c.143C>T in 2 patients (6,12). Three variants, 1 in the noncoding sequence—c.∗464G>A (6)—and 2 in the coding sequence—c.1815G>A (2,10) and c.689A>G (2)—were detected in the *MAPT* gene. Segregated variants were seen only in particular subpedigrees and are signed as ∗ in the Table 2. The most “interesting” variants (PhyloP score ≥2 and at least 1 prediction tool evaluating it as pathogenic) are further emphasized in the Discussion section.

## 4 Discussion

Some past studies have suggested that people living in rural areas could be more exposed to putative environmental, mainly agricultural, influences and would be more likely to develop parkinsonism than those living in urban areas.^[19]^ However, the area surveyed in our study is surrounded by a much larger rural area, in which the agricultural and environmental conditions are quite similar; to date, no signs of higher parkinsonism incidence in this (or any other) part of Central Europe have been reported. A genetic origin of familial parkinsonism thus seems to be the more probable explanation of presence in this population.

The molecular-genetic DNA analysis performed in 12 clinically positive probands in our pedigree has found several new variants in the loci associated with familial parkinsonism in the past:

### 4.1 NM_004562.2: c.1180 C>T *PRKN (PARK2)*

The missense substitution was evaluated as pathogenic using prediction programs (in SIFT pathogenic; in PolyPhen-2 probably pathogenic). It leads to an exchange of acidic for uncharged polar amino acid. Rare occurrence and also a higher degree of evolutionary conservation of the site has been reported. Moura et al^[20]^ identified the variant in 9 (6.8%) of his patients; it was evaluated as a nonpathogenic polymorphism. Lücking et al^[21]^ reported a statistically significant difference between patients and controls: the variant was found in 10/194 patients and in 12/125 controls. In another recent study, this variant was found in similar proportions in both patients and controls.^[22]^

### 4.2 NM_198578.3: c.6241A>G *LRRK2*(*PARK8*)

The substitution is located in an evolutionarily conserved region, which could, according to Polyphen-2, have a pathogenic character. However, SIFT evaluated this variant as benign. Its benign role is supported by the recent finding of Benitez et al,^[23]^ who found this variant in 17 of 478 PD patients and 15 of 337 controls. In another study, Foo et al^[24]^ found the variant in none of the patients and in 1 control (0.25%).

### 4.3 NM_198578.3: c.4541G>A *LRRK2* (*PARK8*)

This missense variant in the coding region leads to the exchange of alkaline amino acid for polar uncharged in the phylogenetically conserved region. According to SIFT, it was evaluated as benign; according to PolyPhen-2, it was evaluated as probably pathogenic. Because of its rare occurrence in the human population and the high phylogenetic conservation of the region, it presumably influences the protein function. On the other hand, Nichols et al^[25]^ described this variant as probably nonpathogenic. Toft et al^[26]^ found the variant in 1 large kindred but they did not confirm its full segregation with parkinsonism. An analysis of the case-control series did not confirm an increased risk of parkinsonism with this variant, and no association with PD has been reported.

### 4.4 NM_198578.3: c.1898C>T *LRRK2 (PARK8)*

This is a missense variant in a coding conserved region, and it leads to the exchange of uncharged polar for nonpolar amino acid. It was evaluated as pathogenic (SIFT and PolyPhen-2). A pathogenic character can thus be expected, but no association with PD has been reported so far.

### 4.5 NM_001123066.3: c.689A>G *MAPT* (*TAU*)

This is a missense variant which leads to the substitution of uncharged polar amino acid for alkaline. The site is evolutionarily conserved. The variant was predicted as pathogenic. Jin et al^[27]^ studied variants of *MAPT* associated with Alzheimer disease in the Spanish population. The variant was identified in 18 patients (10% probands) and 8 controls (5.8%). An association between the variant and the level of tau protein was described in the cerebrospinal fluid.^[28]^ The variant has not yet been functionally analyzed.

The detailed study of the presence of mutual variants in the researched pedigree indicates that the genetic background of this endemic neurodegenerative disease has a clearly heterogenous character: 31 different variants were found in 12 genes. When we tracked the variants within the pedigree, we found a dominant trait in only 3 branches of the family and only for some variants. In patients 3 and 11, we found similar variants of the *EIF4G1* gene. In contrast, *LRRK2* variants were present in the maternal proband only. In patients 6 and 12, we found similar variants of *ADH1C*, *LRRK2*, and *PRKN*; *MAPT* variants were present in the maternal proband only. In patients 2 and 10, similar variants of *PRKN*, *PLA2G6, VPS35*, and *MAPT* were found. Variants of *EIF4G1* and *LRRK2* were present in the maternal proband only, while the variants of *PINK1, DJ-1*, and *GIGYF2* were found only in her daughter. In other pedigree branches, we did not find any similarity in the variant presence in consanguineous relatives. Though many other genes could potentially be associated with PD; we focused on the most common candidate ones.

## 5 Conclusion

Our study has provided an evidence that the recently described endemic neurodegenerative parkinsonism, which can manifest in different phenotypes (see Table 1), has also different genetic background and apparently heterogenous traits. In this molecular–genetic study of the core pedigree, we have not found one “founder” pathogenic variant associated with parkinsonism in all or the most PD patients of this pedigree. Therefore, it could rather be assumed that the familial occurrence of this disease is caused by the combined influence of several “small-effect” genetic variants that accumulate in the population with long-lasting inbreeding behavior. Whether there is any link to the *VPS35* positive familial parkinsonism, which has been discovered in the relatively near region of Upper Austria by Zimprich et al^[29]^ and Struhal et al^[30]^ remains open for further research. For the planned functional analyses, we have so far collected only one brain specimen from the research pedigreee.^[31]^ Therefore, our future study will also utilize proper cell lines with combinations of targeted mutagenesis to assess the effects of particular rare variants.

## Acknowledgments

The authors are grateful to Jan Pavlik, MD, native of Kuzelov and Hornacko researcher, for his substantial contribution to the manuscript by providing ethnographic data and references and to Jarmila Tomesova, the nurse of the general practitioner's office for her contribution to organizing research in the examined region.

## Author contributions

**Conceptualization:** Katerina Mensikova, Radek Vodicka, Radek Vrtel, Marek Godava, Martin Bares, Vladimir Janout, Martin Prochazka, Petr Kanovsky.

**Formal analysis:** Kristyna Kolarikova, Radek Vodicka.

**Investigation:** Tereza Bartonikova, Katerina Mensikova, Kristyna Kolarikova, Radek Vodicka, Pavel Otruba, Michaela Kaiserova, Miroslav Vastik, Lenka Mikulicova, Josef Ovecka, Ludmila Sachova, Frantisek Dvorsky, Jiri Krsa, Petr Jugas, Marek Godava, Martin Bares.

**Methodology:** Katerina Mensikova, Kristyna Kolarikova, Radek Vodicka, Radek Vrtel, Marek Godava, Martin Bares, Vladimir Janout, Petr Kanovsky.

**Supervision:** Radek Vrtel, Martin Prochazka, Petr Kanovsky.

**Writing – original draft:** Tereza Bartonikova, Kristyna Kolarikova, Radek Vodicka.

**Writing – review & editing:** Katerina Mensikova, Petr Hlustik, Martin Prochazka, Petr Kanovsky.
